# Impact and cost-effectiveness of strategies to prevent respiratory syncytial virus (RSV) disease in Vietnam: A modelling study

**DOI:** 10.1016/j.vaccine.2023.09.003

**Published:** 2023-11-02

**Authors:** Lien Anh Ha Do, Nguyen Thanh Nhan Le, Sarwat Mahmud, Kim Mulholland, Clint Pecenka, Andrew Clark

**Affiliations:** a New Vaccines Group, Murdoch Children’s Research Institute, Melbourne, Australia; b Department of Pediatrics, The University of Melbourne, Melbourne, Australia; c Children’s Hospital 1, Ho Chi Minh City, Viet Nam; d Department of Health Services Research and Policy, London School of Hygiene and Tropical Medicine, London, United Kingdom; e Department of Infectious Disease Epidemiology, London School of Hygiene and Tropical Medicine, London, United Kingdom; f PATH, Seattle, USA

**Keywords:** Respiratory syncytial virus (RSV), Low-income and middle-income countries (LMICs), Maternal vaccine, Monoclonal antibody, Cost-effectiveness analysis

## Abstract

**Background:**

New prevention strategies for respiratory syncytial virus **(**RSV) are emerging, but it is unclear if they will be cost-effective in low- and middle-income countries. We evaluated the potential impact and cost-effectiveness of two strategies to prevent RSV disease in young children in Vietnam.

**Methods:**

We used a static cohort model with a finely disaggregated age structure (weeks of age <5 years) to calculate the RSV disease burden in Vietnam, with and without a single dose of maternal vaccine (RSVpreF, Pfizer) or of monoclonal antibody (Nirsevimab, Sanofi, Astra Zeneca). Each strategy was compared to no pharmaceutical intervention, and to each other. We assumed both strategies would be administered year round over a ten-year period. The primary outcome measure was the cost per disability-adjusted life year (DALY) averted, from a societal perspective. We ran probabilistic and deterministic uncertainty analyses.

**Results:**

With central input assumptions for RSVpreF vaccine ($25/dose, 69 % efficacy, 6 months protection) and Nirsevimab ($25/dose, 77 % efficacy, 5 months protection), both options had similar cost-effectiveness ($3442 versus $3367 per DALY averted) when compared separately to no pharmaceutical intervention. RSVpreF vaccine had a lower net cost than Nirsevimab (net discounted cost of $213 m versus $264 m) but prevented fewer RSV deaths (24 % versus 31 %). Our results were very sensitive to assumptions about the dose price, efficacy, and duration of protection. At $5/dose and a willingness-to-pay threshold of 0.5 times the national GDP per capita, both prevention strategies have the potential to be cost-effective.

**Conclusions:**

RSVpreF vaccine and Nirsevimab may be cost-effective in Vietnam if appropriately priced.

## 1 Introduction

Acute lower respiratory infections (ALRIs) are the leading cause of mortality in children younger than five years of age worldwide [1], with respiratory syncytial virus (RSV) being the most important pathogen [2]. Most of the deaths associated with RSV occurred in children less than 6 months of age living in low- and middle-income countries (LMICs) [2].

In 2017 in Vietnam, ALRIs were estimated to cause 13 % of deaths in children <5 years [3]. In southern Vietnam and in central Vietnam, RSV was also the leading pathogen in a population-based ALRI surveillance study of children younger than two years of age [4], [5], [6].

Palivizumab (AstraZeneca), a monoclonal antibody (mAb), is the only pharmaceutical intervention currently available to prevent RSV in young children, but it is expensive [7]. Two emerging pharmaceutical strategies have demonstrated high efficacy against severe RSV disease in clinical trials and may be feasible for use in LMICs. A single injection long-acting mAb (*Beyfortus*™, Nirsevimab, Astra Zeneca and Sanofi) has been approved for use in infants in Europe and United States (US) [8], [9] but it is very costly. Meanwhile a maternal vaccine (RSVpreF or PF-06928316, Pfizer) [10] has been approved in the US for use in the third trimester of pregnancy. Both strategies are designed to provide newborns with protection against RSV disease as early in life as possible.

Forthcoming reviews of Nirsevimab and RSVpreF by the World Health Organisation (WHO) Strategic Advisory Group of Experts on Immunisation (SAGE) will have implications for the potential approval and recommended use of both products in LMICs. National decision-makers will also need to assess whether to recommend the introduction of one or both RSV prevention strategies. A preliminary modelling analysis could help synthesise available country-level data, identify key drivers of cost-effectiveness, and establish future data needs. It should also provide a foundation for new analyses to be run in the future as new input data emerges.

This paper provides a preliminary assessment of the potential impact and cost-effectiveness of the infant mAb (Nirsevimab) and the maternal vaccine (RSVpreF) strategies in Vietnam.

## 2 Methods

### 2.1 Modelling approach

We used version 1.6 of the universal vaccine decision-support model, UNIVAC, [11] to evaluate the potential impact and cost-effectiveness of introducing Nirsevimab and RSVpreF over a ten-year period (2025–2034) in Vietnam. Without detailed estimates of the national seasonal RSV incidence, we assumed both strategies would be administered year-round. UNIVAC is an Excel static proportionate outcomes cohort model with a finely disaggregated age structure (weeks of age for children <5 years). The UNIVAC-RSV model is described in detail elsewhere [12]. In brief, UNIVAC is populated with the United Nations (UN) (2022 revision) estimates of the number of individuals alive in each single calendar year and single year of life in Vietnam [13]. For each birth cohort, numbers of life-years experienced between birth and age five years are multiplied by rates of severe RSV disease outcomes (cases, clinic visits, hospital admissions and deaths) and non-severe RSV disease outcomes (cases, clinic visits). Rates are entered per 100,000 per year in children aged <5 years. The UNIVAC model calculates the numbers of cases, clinic visits, hospital admissions, deaths, and disability-adjusted life years (DALYs) expected to occur with and without each of the two RSV interventions over the lifetimes of each birth cohort.

The expected numbers of disease outcomes aged <5 years are assigned to weeks of age <5 years. For each RSV prevention strategy, the percent reduction in each disease outcome is calculated for each week of age by multiplying intervention coverage in the relevant week of age by the assumed efficacy against that outcome in the relevant week of age. For simplicity, all disease outcomes are assumed to be independent, hence if a severe RSV case has an outpatient clinic visit and then later goes to inpatient care, this case is included in both the rate of clinic visits and the rate of hospital admissions.

The primary outcome measure is the cost (US$) per DALY averted from the societal perspective, accounting for all costs and benefits aggregated over the ten birth cohorts (2025–2034). All future costs and health benefits were discounted at 3 % per year, and all costs represent 2022 US$ (1US$ currency exchange rate to Vietnam Dong = 23,195).

Vietnam does not have a strict willingness-to-pay (WTP) threshold for determining if an intervention is cost-effective. In this study, we calculated the probability the vaccine would be cost-effective at 0.25, 0.5, and 1 times the national gross domestic product (GDP) per capita [14]. These are broadly consistent with the 0.26–0.89 range estimated by Ochalek et al for Vietnam [15] based on a range of different approaches for estimating the health effects of changes in health expenditure.

All inputs assumed for Vietnam are summarised in Table 1.

**Table 1:** Input parameters used to evaluate the impact and cost-effectiveness of RSV prevention strategies in Vietnam for 10 birth cohorts (period 2025–2034).

**Input parameter**	**Value**	**Uncertainty range**	**Source**
**RSV disease event rate per 100,000 per year (<5yrs)**			
RSV (non-severe) cases	3,220	2,610–3,850	Yoshida, 2013 [16] minus severe
RSV (non-severe) visits	2,126	1,724–2,543	WHO (66 % access to care) [17]
RSV (severe) cases	1,400	800–2,420	Li et al., 2022 [2]
RSV clinic visits	925	538–1,598	WHO (66 % access to care) [17]
RSV hospital admissions	644	368–1,138	New analysis (Ho Chi Minh City)
RSV deaths	20	15–27	Li et al., 2022 [2]
Percentage of RSV non-severe cases by age *			
<1 month	0 %	N/A.	Li et al., 2022 [2]
<3 months	7 %		
<6 months	35 %		
<9 months	57 %		
<12 months	70 %		
<2 years	90 %		
<5 years	100 %		
Percentage of RSV severe cases by age **			
<1 month	2 %	N/A.	New analysis of RSV-ALRI hospital admissions in Ho Chi Minh City (see methods).
<3 months	25 %		
<6 months	51 %		
<9 months	64 %		
<12 months	72 %		
<2 years	87 %		
<5 years	100 %		
**DALY weights**			
RSV non-severe cases	0.051	0.032–0.074	GBD, 2019 [20]
RSV severe cases	0.133	0.088–0.190	GBD, 2019 [20]
**Duration of illness (days)**			
RSV non-severe cases	5	3–7	Hall et al. [19]
RSV severe cases	7	3–22	New analysis (Ho Chi Minh City)
**Cost per visit/admission (US$)**			
RSV clinic visits	52	32–85	Do et al. BMC Infect Dis 2023 [21]
RSV hospital admissions	165	95–249	Do et al. BMC Infect Dis 2023 [21]
**Impact of RSV mAb (infant)**			
Program coverage ***	88 %	79 –97 %	BCG, MICS report [27], [28]
Efficacy (RSV non-severe cases)	75 %	50–87 %	AstraZeneca Press release [30]
Efficacy (RSV severe cases)	77 %	50–90 %	AstraZeneca Press release [30]
Duration of protection (months)	5	4-6	AstraZeneca Press release [30]
**Impact of RSV vaccine (maternal)**			
Program coverage	70 %	63–77 %	ANC, Baral et al, 2020 [26]
Efficacy (RSV non-severe cases)	51 %	29–67 %	Kampmann et al. [29]
Efficacy (RSV severe cases)	69 %	44–84 %	Kampmann et al. [29]
Duration of protection (months)	6	3-7	Kampmann et al.[29]
**Assumed for both infant mAb and maternal vaccine**			
**Percentage wastage**			
RSV intervention doses	5 %	2–8 %	Assumption
Syringes	5 %	2–8 %	Assumption
Safety boxes	5 %	2–8 %	Assumption
**Costs (US$)**			
Price per dose	$25	$5–$25	
Syringe price per dose (US$)	$0.0278	N/A	UNICEF Supply Div. [24]
Safety box price per dose (US$)	$0.0121	N/A	UNICEF Supply Div. [24]
International handling (% of price per dose)	1 %	N/A	UNICEF Supply Cat. [22]
International delivery (% of price per dose)	6 %	2–15 %	Debellut et al. [23]
Incremental health system cost per dose (US$)	$2.02	N/A	ICAN [25]

**Fig. 1:**
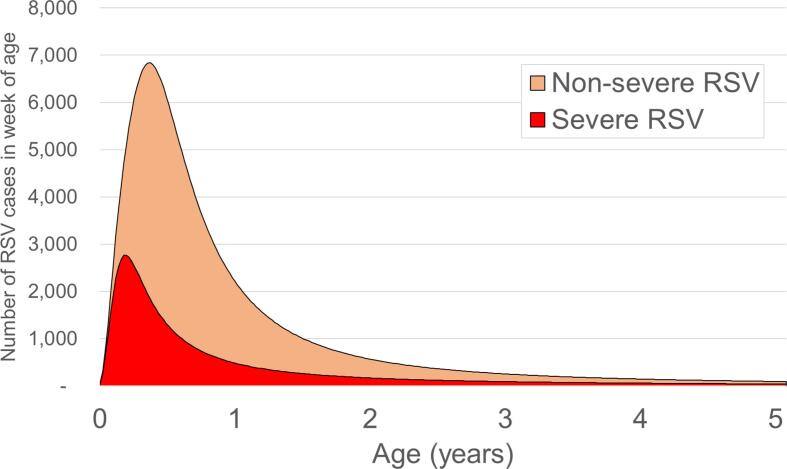
Estimated age distribution of severe and non-severe RSV-ALRI cases in Vietnam. Caption: the parametric Burr distribution had the most favourable root mean square error (RMSE) compared to other standard distributions e.g., Log logistic. The Burr distribution (Burr type XII) has shape 1 (γ), shape 2 (α), and scale (θ), all of which must be positive values. Parameters for the severe disease age distribution were: shape 1 = 2.7, shape 2 = 0.2, scale = 9.8. Parameters for the non-severe age distribution were: shape 1 = 3.2, shape 2 = 0.4, scale = 21.4. The cumulative distribution function (cdf) of the Burr distribution (for *x* weeks of age) is:.$f\left(x\right)=1-{\left[1+{\left(\frac{x}{\theta }\right)}^{\gamma }\right]}^{-\alpha }$

* Non-severe age distributions are assumed for RSV clinic visits by non-severe cases. A Burr distribution (found in UNIVAC model step 2 of inputs page) was used to calculate the age distribution by week of age (Fig. 1).

**Severe age distributions are also assumed for RSV hospital admissions, RSV deaths, and RSV clinic visits. A Burr distribution (found in UNIVAC model step 2 of inputs page) was used to calculate the age distribution by week of age (Fig. 1).

***Coverage was 88 % based on BCG coverage at 52 weeks. Lower coverage was assumed in earlier weeks of age using timeliness data from the 2014 MICS.

WHO: World Health Organisation; GBD: Global Burden Diseases; BCG: Bacillus Calmette–Guérin vaccine; MICS: Multiple Indicator Cluster Survey; ANC: antenatal care; UNICEFF: United Nations International Children's Emergency Fund; ICAN: Immunization Costing Action Network.

### 2.2 Disease burden

A study in Nha Trang by Yoshida et al. [16] estimated 4,620 (95% CI 3,410–6,270) RSV-ALRI cases per 100,000 per year in children aged <5 years. We estimated 1,400 (95% CI 800–2,420) severe RSV-ALRI cases (e.g. “with chest wall indrawing”) per 100,000 per year in children aged <5 years based on a systematic review and meta-analysis of LMICs by Li et al [2]. We estimated the rate of non-severe RSV-ALRI cases by subtracting the severe RSV-ALRI rate from the total RSV-ALRI rate (Table 1).

We estimated 20 RSV-ALRI deaths per 100,000 per year in children aged <5 years using estimates from Li et al. for the same income stratum e.g lower-middle income countries [2]. This is equivalent to around 5 % of all under-five deaths in Vietnam.

We assumed that 66 % of RSV-ALRI cases would be associated with a clinic visit based on WHO estimates of the mean percentage of children <5 years with pneumonia symptoms who were taken to a healthcare provider in lower-middle income countries [17].

We estimated the annual rate of RSV-ALRI hospital admissions to be 644 (95% CI 368 – 1138) per 100, 000 per year in children aged <5 years by combining several local data sources. First, we calculated the number of ALRI admissions (ICD-10 codes J-12, J20-22, and J18) in children less than 2 years of age attending a referral paediatric hospital in Ho Chi Minh City (Children’s Hospital 1) over a two-year period (2018–2019). Second, we applied age-specific estimates of the fraction of ALRIs attributable to RSV based on two previous studies conducted in Ho Chi Minh City [4], [5]. Third, we fit a parametric (Burr) age distribution to the derived counts of RSV-ALRI hospital admissions in children less than 2 years of age and extrapolated the curve to estimate the number of cases expected to occur between 2 and 5 years of age (Fig. 1). Finally, we assumed a hospital population catchment size of 5,941,573 children <5 years by multiplying the entire population in the catchment area (72,563,300, provided by the local administration) by the percentage of the total population of children <5 years (8 %) estimated by UN world population prospects (UNWPP) [18]. The RSV-ALRI admission rate estimated using this method (644 per 100,000 children <5 years) was consistent with the estimate by Li *et al.* for LMICs (610 per 100,000 children <5 years) [2].

The age distribution calculated for RSV-ALRI hospital admissions was applied to all severe RSV disease outcomes. To calculate the age distribution for non-severe RSV disease outcomes, we estimated the number of severe RSV-ALRI cases expected to occur in the year 2019 in broad age groups (<3m, 3–5 m, 6–11 m, 12–59 m) and assumed that 27 %, 74 %, 76 %, and 71 % would be non-severe. These percentages were calculated by adjusting age-specific incidence rates from Li *et al.* [2] for LMICs to make them consistent with the overall rate of RSV-ALRI estimated in Nha Trang by Yoshida et al [16]. We then fit a parametric (Burr) age distribution to the estimated counts of non-severe RSV-ALRI disease cases in children <5 years (Fig. 1).

When calculating DALYs, we assumed non-severe RSV-ALRI symptoms would last for five days based on a previous publication [19]. We assumed seven days for severe RSV-ALRI symptoms based on data from Children’s Hospital 1 (2018–2019) and two previous studies [4], [5]. We assumed disability weights of 5.1 % and 13.3 % for non-severe and severe RSV-ALRI disease cases, respectively, based on GBD 2019 DALY weights for moderate and severe lower respiratory infections [20]. We used UN estimates life-expectancy at specific ages to determine premature mortality averted [13]. For example, life expectancy at age 1 year was 70 years for males and 79 years for females in the 2025 birth cohort.

### 2.3 Cost of clinic visits and hospital admissions

We estimated US$ 52 (IQR 32–85) per RSV clinic visit and US$ 165 (IQR 95–249) per RSV hospital admission based on a prospective study of children aged <2 years who sought care at a major paediatric referral hospital in southern Vietnam between September 2019 and December 2021. The societal costs in Table 1 include direct medical costs (e.g., hospitalisation billing), non-medical costs (e.g., transportation, accommodations, etc.), and indirect costs (e.g., the opportunity cost of missed work) incurred prior to admission, during the hospitalisation, or during medical visits and after discharge [21].

### 2.4 RSV prevention strategy costs

The dose price of each intervention is highly uncertain, so we used $25/dose for the base-case analysis (Table 1) and ran separate scenarios assuming $5 and $15 per dose. Costs for international handling [22] and delivery [23], safety boxes, and syringes were taken from reference sources [24]. We assumed wastage of 5 % for doses, syringes, and safety boxes (Table 1). The percent wastage is converted into a factor [1/ (1 −  % wastage)] which is multiplied by the expected number of doses required to meet the anticipated level of coverage.

The health system delivery costs associated with RSV interventions are uncertain and adaptations to existing immunization delivery platforms may be required. The incremental health system delivery cost per dose will include the costs of additional training, transportation, cold-chain expansion etc. Given the lack of empirical cost of delivery of these interventions, we derived the cost estimates from existing literature, the Immunization Delivery Cost Catalogue (IDCC) repository. Specifically, a health system cost of delivery of US$ 2.02 per immunization, an average estimate for the LMIC was used [25]. (Table 1).

### 2.5 RSV prevention strategy impact

We assumed maternal vaccination could achieve 70 % coverage of pregnant women each year based on an estimate from Baral et al [26]. This represents the proportion of pregnant women who attended at least one antenatal care (ANC) visit at 24–36 weeks of gestation, using data from the 2002 Demographic and Health survey (DHS) in Vietnam [26].

We assumed infant mAbs could achieve the same coverage and timeliness (age-specific coverage) as reported for Bacillus Calmette–Guérin (BCG) vaccine in the 2014 Multiple Indicator Cluster Survey (MICS) in Vietnam. We included coverage in each week of age that corresponds to 58 %, 82 %, and 88 % by 1, 3, and 12 months of age respectively [27], [28]. We assumed year-round RSV disease incidence and year-round mAb administration, so any doses given later in infancy were still assumed to have some effect on incident cases that occur later in infancy.

Efficacy estimates were taken from November 2022 press releases of clinical trials for both the maternal vaccine (RSVpreF) [29] and infant mAb (Nirsevimab) [30] (Table 1). RSVpreF efficacy was reported after three and six months after birth [29]. For Nirsevimab, the efficacy was reported after five months and involved combining evidence from the Phase 3 MELODY trial and the Phase 2b trial [30]. The end points were RSV ALRI cases that were medically attended (used as a proxy for efficacy against non-severe RSV cases and clinic visits) and RSV-ALRI cases that were hospitalised (used as a proxy for efficacy against severe RSV cases, clinic visits, hospital admissions and deaths). In the base case we assumed these efficacy values would be fixed for the duration of follow-up in the clinical trials and then fall to zero thereafter. We also ran alternative efficacy and waning scenarios in uncertainty analyses.

### 2.6 Uncertainty analysis

For both RSV intervention strategies, we ran the following alternative scenarios:

(i) *Efficacy = fitted gamma.* For maternal vaccination, cumulative efficacy (cE) was reported to be 81.8 % at three months and 69.4 % at six months of age. We used previously described methods [12] to calculate the instantaneous efficacy (iE) at each week of age. This assumes very high initial efficacy followed by a gradual ebbing of protection that approaches zero by 12 months. We assumed a pooled age distribution of RSV disease from six LMICs and assumed that iE could not become negative. A similar method was used for infant mAb.
(ii) *Efficacy = 3 month duration.* For maternal vaccination we assumed efficacy of 81.8 % against severe RSV disease and 57.1 % against non-severe RSV disease for a three-month period, and zero protection thereafter. This scenario was not applicable to infant mAb.
(iii) *Price reduced to $15 per dose.*
(iv) *Price reduced to $5 per dose*.

Sensitivity analyses were also conducted to identify parameters with the most influence on cost-effectiveness. Each parameter’s central estimate was varied in turn by +/−10 % and the change in cost per DALY averted was noted.

We also ran a probabilistic uncertainty analysis to indicate the range of parameter uncertainty around the incremental costs and benefits (DALYs averted). In the absence of good quality information on the correlation structure and distribution shapes for each input parameter, we assumed simple PERT-Beta distributions [31] informed by the ranges and most likely values outlined in Table 1. We ran 1000 Monte-Carlo simulations for each RSV prevention strategy and each fixed dose price scenario ($5, $15, $25). The results from this analysis were used to generate cost-effectiveness acceptability curves (CEACs) showing the probability that each intervention would be cost-effective at different willingness-to-pay (WTP) thresholds.

## 3 Results

### 3.1 Lifetime costs and effects based on central input assumptions

Table 2 summarises the results based on our central input assumptions for RSVpreF vaccine ($25/dose, 69 % efficacy, 6 months protection) and Nirsevimab ($25/dose, 77 % efficacy, 5 months protection). Nirsevimab was estimated to be more costly than RSVpreF vaccine (net discounted cost of about $303 versus about $243 million) but more impactful. Nirsevimab could avert more severe RSV cases in children <5 years than RSVpreF vaccine (e.g., 279,329 versus 220,992) and avert more RSV deaths than RSVpreF vaccine in children <5 years (2,965 versus 2,345, equivalent to an RSV mortality reduction of 31 % versus 24 %).

**Table 2:** Lifetime costs and effects of maternal vaccination ($25/dose, 69 % efficacy, 6 months protection) and infant mAb ($25/dose, 77 % efficacy, 5 months protection) in Vietnam over the period 2025–2034.

	**Do nothing**	**Maternal vaccine** **(RSVpreF)**	**Infant mAb** **(Nirsevimab)**
**Lifetime costs and effects**			
Non-severe RSV cases <5 yrs	2,091,154	1,780,365	1,632,101
Non-severe RSV clinic visits <5 yrs	1,380,998	1,175,753	1,077,840
Severe RSV cases <5 yrs	909,197	688,205	629,869
Severe RSV clinic visits <5 yrs	600,434	454,490	415,965
Severe RSV hospital admissions <5 yrs	418,465	316,751	289,902
Severe RSV deaths <5 yrs	9,650	7,304	6,685
DALYs (discounted*)	251,186	189,305	172,929
Vaccine program costs (discounted*)	$0	$243,431,891	$303,921,547
Societal healthcare costs (discounted*)	$147,268,288	$116,859,088	$106,858,120
**Differences (comparator = no vaccine)**			
Non-severe RSV cases <5 yrs	–	310,789	459,052
Non-severe RSV clinic visits <5 yrs	–	205,245	303,158
Severe RSV cases <5 yrs	–	220,992	279,329
Severe RSV clinic visits <5 yrs	–	145,943	184,469
Severe RSV hospital admissions <5 yrs	–	101,713	128,563
Severe RSV deaths <5 yrs	–	2,345	2,965
Percent reduction in severe RSV deaths <5 yrs		24 %	31 %
DALYs (discounted*)	–	61,882	78,258
Vaccine program costs (discounted*)	–	$243,431,891	$303,921,547
Societal healthcare costs (discounted*)	–	-$30,409,200	-$40,410,168
**Cost (US$) per DALY averted** **(comparator = no vaccine)**			
Cost (discounted*)	–	$213,022,691	$263,511,379
DALYs averted (discounted*)	–	61,882	78,258
Cost per DALY averted (discounted*)	–	$3,442	$3,367
Cost (US$) per DALY averted (comparator = least costly non-dominated** option)			
Cost (discounted*)	–	$213,022,691	$50,488,688
DALYs averted (discounted*)	–	61,882	16,376
Cost per DALY averted (discounted*)	–	$3,442	$3,083
GDP per capita (2022)	–	$3,694	$3,694
Cost per DALY averted (discounted*) (expressed as a % of GDP/capita)	–	93 %	83 %

*Future costs/effects were discounted at a rate of 3 % per year.

** Dominated options have higher costs and lower benefits than alternative options. The least costly non-dominated option is maternal RSV vaccination. This is used as the comparator for Infant mAb when calculating the incremental cost-effectiveness of mAb versus maternal vaccination.

Nirsevimab was slightly more cost-effective than RSVpreF vaccine ($3,367 versus $3,442 per DALY averted) when each intervention was compared to no pharmaceutical intervention. When we compared Nirsevimab to RSVpreF directly, the incremental cost per DALY averted for Nirsevimab was $3,083 which is 83 % of the national GDP per capita in Vietnam.

Around 99 % of the DALYs averted were attributed to premature mortality averted highlighting the important contribution of RSV mortality rather than morbidity to overall DALYs. Clinic visits were associated with around half the total societal healthcare costs averted by both interventions.

### 3.2 Cost-effectiveness at different willingness-to-pay thresholds

**Fig. 2:**
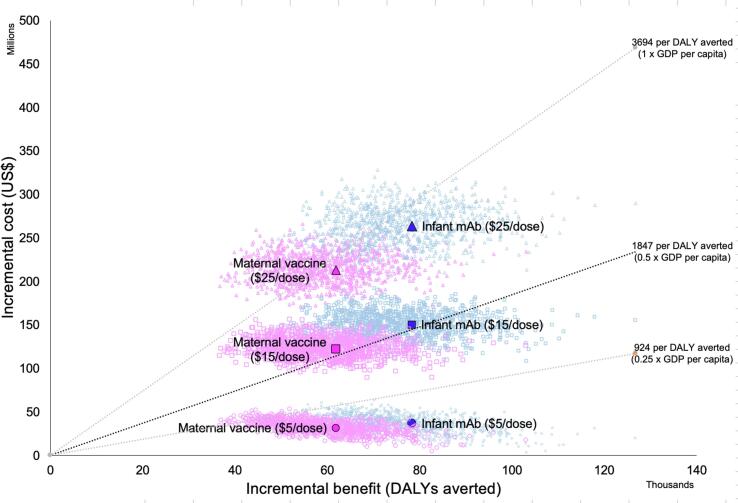
Probabilistic clouds of incremental costs and benefits of both RSV prevention strategies assuming three different dose prices ($5, $15, and $25).

Fig. 2 shows incremental costs and benefits of both RSV prevention strategies assuming three different dose prices ($5, $15, and $25). Probabilistic uncertainty clouds represent 1000 runs per scenario and indicate the range of parameter uncertainty around the central estimates. Three WTP lines (0.25, 0.5, and 1.0 times the national GDP per capita) indicate the dose price that may be acceptable at different WTP thresholds. At all prices, the infant mAb is assumed to generate greater incremental health benefits than maternal vaccination, though at a slightly higher net incremental cost. At a WTP threshold of 1-times the national GDP per capita a dose price of less than $15 would have a high probability of being cost-effective (probabilistic clouds are entirely below the WTP threshold line). However, should the WTP threshold be lower (e.g., 0.25 times the national GDP per capita), the dose price would need to be less than $5/dose to have a high probability of being cost-effective.

**Fig. 3:**
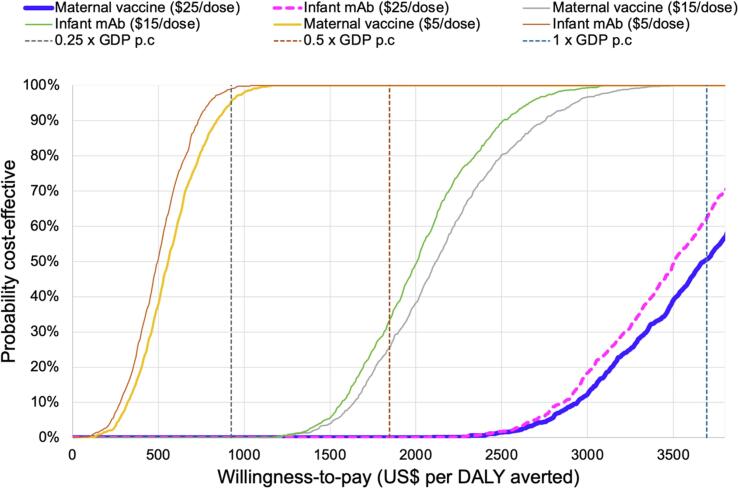
Probability that each RSV prevention strategy (monoclonal antibody or maternal RSV vaccine) will be cost-effective compared to no intervention, at different WTP thresholds.

Fig. 3 shows the probability that each strategy will be cost-effective compared to no intervention, at different WTP thresholds. At a WTP threshold of 0.25 times the national GDP per capita, the RSV prevention strategy with the most favourable cost-effectiveness would need to be priced at $5/dose or less to achieve a greater than 95 % probability of being cost-effective.

### 3.3 Scenario and sensitivity analysis

**Fig. 4:**
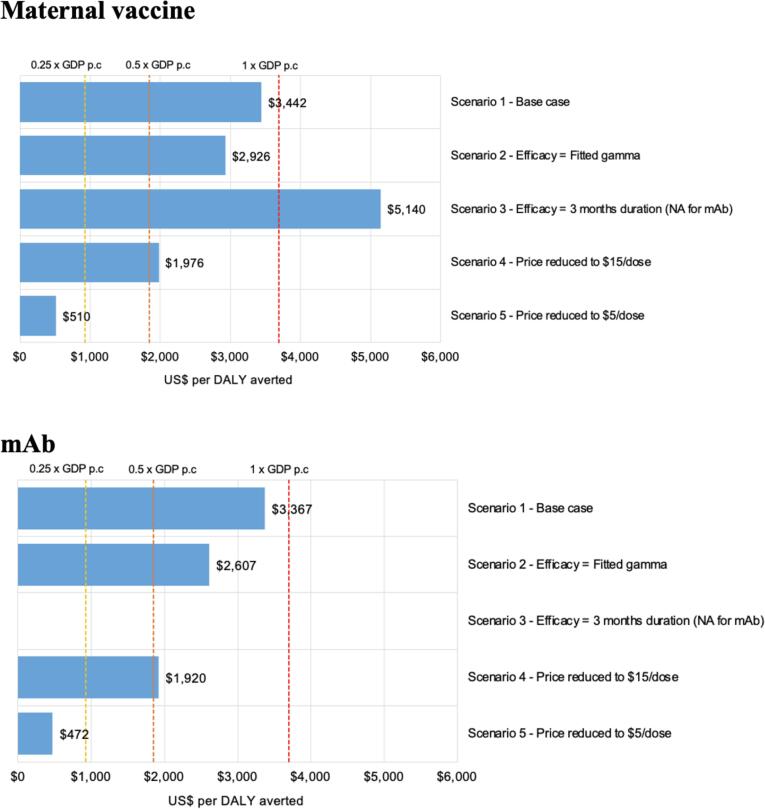
Cost per DALY averted (US$) for several alternative scenarios for maternal vaccine and mAb.

Fig. 4 shows the robustness of the cost-effectiveness results by recalculating cost-effectiveness for a range of alternative deterministic ‘what-if’ scenarios. Three potential WTP thresholds (at 0.25 times, 0.5 times, and 1 times of the national GDP per capita) were used.

**Fig. 5:**
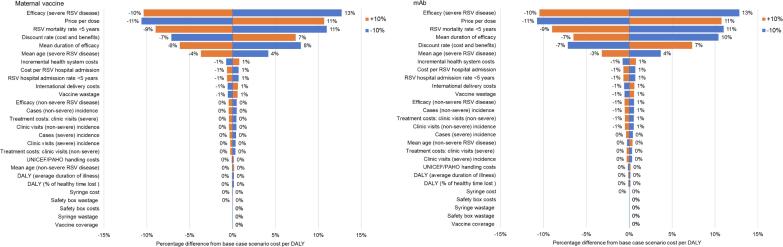
Difference in cost per DALY averted (%) relative to baseline cost of $3,442 for maternal vaccine and $3,367 for mAb, when each parameter is varied by +/-10 %.

For maternal vaccination (RSVPreF), if the duration of protection is only three months (not applicable for Nirsevimab), the cost per DALY averted is unfavourable (higher than a WTP threshold of 1 times of the national GDP per capita). In scenario 2 (fitted gamma with protection gradually waning over time) the cost per DALY averted is more favourable than the base case scenario for both maternal vaccine and mAb. In the scenarios where the price was reduced, the cost per DALY averted only became less than 0.25 times of the national GDP per capita when the dose price was $5/dose. The parameters with the greatest influence on the cost-effectiveness results were similar for maternal vaccine and mAb. Results were very sensitive to the changes in efficacy, dose price, RSV mortality rate, duration of protection, discount rate, and age distribution of severe RSV disease (Fig. 5).

## 4 Discussion

In this paper we present a preliminary assessment of the cost-effectiveness of two strategies for preventing RSV disease in young children in Vietnam. Using plausible assumptions about the expected costs, health impact, and healthcare costs averted by each strategy, we find that RSVPreF and Nirsevimab each have the potential to be cost-effective in Vietnam, if appropriately priced. Our analysis suggests the RSV prevention strategies would need to be priced at less than $5/dose in order to satisfy the lowest (most pessimistic) estimate of WTP recently determined by Ochalek et al for Vietnam (cost per DALY averted less than 0.25 times the national GDP per capita) [15]. With our central input assumptions, we find that Nirsevimab could be more costly and more impactful than RSVPreF, but both options would have a similar cost per DALY averted compared to no pharmaceutical RSV intervention. The higher coverage assumed for Nirsevimab (88 % versus 70 % for RSVPreF) is the main reason it would be more costly and impactful. However, the anticipated coverage of both strategies is highly uncertain, and our coverage assumptions would change substantially if the RSV prevention strategies were restricted to use in specific risk groups.

A strength of our analysis is that we have included new analyses and data that is representative of the Vietnamese context. This includes estimates of the RSV hospital admission rate, the RSV disease age distribution in granular age bands (weeks of age), and costs of RSV illness. Our analysis also includes the latest available evidence on the efficacy and duration of protection associated with both RSVpreF and Nirsevimab. Our analysis should also provide a foundation for new analyses to be run in the future as new input data emerges. We used the transparent and user-friendly UNIVAC decision-support model [12] which could be easily updated and reviewed by local stakeholders, including “non-modellers,” to help increase local capacity and ownership of model results.

Our study is a preliminary assessment and should be updated as new evidence emerges, particularly on the price and duration of protection associated with each strategy. To assess parameter uncertainty, we ran a range of probabilistic and deterministic uncertainty analyses and identified the dose price that might be acceptable at different WTP thresholds. However, we did not assess potentially important uncertainty about the delivery strategy (or coverage) assumed for RSVpreF and Nirsevimab. A substantial part of this uncertainty is our assumption of ‘year-round’ disease incidence and ‘year-round’ administration of RSV prevention strategies. In Vietnam, there is known variability in different regions, and it was not possible to generate reliable estimates of aggregate national seasonality. However, should it be possible to target the interventions and protect infants and young children just prior to each RSV season, then this could result in more favourable cost-effectiveness estimates, assuming the cost of reaching high-risk infants is not prohibitive. Our modelled estimates of health impact also excluded any potential indirect herd immunity benefits. If new evidence emerges that shows maternal vaccines or mAbs will interrupt transmission, it would make our estimates more cost-effective.

We assumed the efficacy of RSVpreF and Nirsevimab would be the same as reported in the November 2022 press releases with the Phase 3 trial results [29], [30]. However, updated assumptions may be needed following a more detailed review of the full published trial results. In addition, while both trials recruited participants from a range of countries, it is unclear how representative the results will be to Vietnam. Most trial sites were in high-income countries where the burden of severe RSV disease is lower than in Vietnam. Our alternative scenarios of efficacy and duration of protection indicated important differences in cost-effectiveness results, emphasizing the need for greater certainty on the efficacy of each intervention and shape of the waning protection curve over time. The duration of protection associated with maternal vaccination could be affected by the gestational age and other factors impacting maternal antibody transfer. In addition, it may not be possible to achieve BCG coverage levels if the vaccine needs to be targeted to those at greatest risk. One approach would be to use both maternal vaccination and infant mAbs in combination, with infant mAbs administered to preterm newborns whose mothers were not previously vaccinated. Our analysis suggests the incremental cost-effectiveness of administering mAb to infants not previously protected by maternal vaccination is likely to be similar to the cost-effectiveness of maternal vaccination, but more data on the risk and coverage of different target groups would be needed to make a more informed assessment.

The majority of DALYs in RSV infections were attributable to premature mortality, with Years of Life Lost representing approximately 99 % of DALYs. The large impact of mortality on DALYs was further indicated in the one-way sensitivity analysis (Fig. 5). Although the mortality rate used in this study was not specific to Vietnam due to the paucity of country-specific data, and instead was income-stratum specific for LMICs as the closest approximation for Vietnam, the impact of mortality is consistent with what would be expected given almost all deaths attributable to RSV occur in LMICs (97 %, Li et al [2]).

Our sensitivity analysis highlighted that the cost-effectiveness results were sensitive to the dose price assumed for each RSV prevention strategy. We estimated a maternal vaccine or mAb at US$5 per dose would cost approximately 0.25 times the national GDP per capita (e.g., $923.5) per DALY averted, in Vietnam. The median private sector dose price of pediatric vaccines currently available in the South of Vietnam is US$ 12 (IQR 8–27), but the private sector dose price is lower (around US$ 5) for vaccines that are also simultaneously used in the National Immunization Program. Our assessment that the dose price may need to be less than $5 per dose is consistent with the conclusion from Li et al that “any prophylaxis would have to be competitively priced to be considered cost-effective in Gavi-eligible settings” [32]. Both UNIVAC and the model used by Li et al are static cohort models, but our estimates of RSV disease burden are based on a more recent systematic review and have been supplemented by a new analysis of hospital data from Ho Chi Min City. We also used recently published disease treatment costs from Vietnam. Our estimates of the efficacy and duration of protection associated with maternal vaccination and infant mAb were also based on updated clinical trial data.

Our estimates of the age distribution of severe RSV disease were derived by combining age-specific estimates of ALRI hospital admissions with age-specific estimates of RSV-positivity from the same study location [4], [5]. One important limitation of this method is that these RSV-positivity studies did not enroll many patients younger than 1 month of age and so we had to assume less than 2 % of severe RSV cases occurred for the less than 1 month of age group [4], [5]. Accurately estimating RSV burden in the under 1 month age group is important for assessing the impact of maternal vaccines and mAbs and warrants more research in LMICs. The estimated costs of RSV disease that we used in our model were in the low range of reported public health care costs in Vietnam. This could be explained by the fact that the costs associated with outpatient clinic visits are mostly covered by the patient’s family (out-of-pocket), while any children admitted to hospital due to an emergency, or formally referred to a tertiary public hospital is eligible to receive 80 % support from the National Health Insurance System for approved medications and treatment.

To the best of our knowledge, our study is the first analysis to assess the cost-effectiveness of two forthcoming RSV prevention strategies in Vietnam and indicates that both RSVpreF vaccine and Nirsevimab may be cost-effective if appropriately priced. Our preliminary analysis has identified important priorities for data collection and should be updated as new input data emerges.

## Consents and ethics approvals

Not applicable.

## Consent for publication

Not applicable.

## Availability of data and materials

The majority of input parameters were derived from public domain resources. Local data are available from the corresponding author, upon reasonable request.

## Authors' contributions

LAHD coordinated the study, collected data, performed the data analysis, and drafted the manuscript; NTNL coordinated the study, collected data, and provided critical revision of the manuscript; SM performed data analysis and provided critical revision of the manuscript; KM provided expertise for the study models development and provided critical revision of the manuscript; CP was responsible for project overview, obtained the funding for the project, and provided critical revision of the manuscript; AC conceptualized the models and the study design, developed the models, provided expertise on development of all study materials and manuscript, reviewed data analysis, and provided critical revision of the manuscript.

All authors read and approved the final manuscript.

## Funding

This work was supported, in whole or in part, by the Bill & Melinda Gates Foundation [Grant Number INV-007610]. Under the grant conditions of the Foundation, a Creative Commons Attribution 4.0 Generic License has already been assigned to the Author Accepted Manuscript version that might arise from this submission. The funder had no role in the study design, data collection, data analysis, data interpretation, writing of the report, or the decision to submit.

## Declaration of Competing Interest

The authors declare the following financial interests/personal relationships which may be considered as potential competing interests: Lien Anh Ha Do reports financial support was provided by Bill & Melinda Gates Foundation. Prof. Kim Mulholland is currently member of WHO SAGE, he was a member of DSMB for Novavax COVID-19 vaccine without payment.

## Data Availability

Majority of input parameters data derived from public domain resources. Local data are available from the corresponding author, upon reasonable request.
